# Role of the PD‐1/PD‐L1 Signaling in Multiple Sclerosis and Experimental Autoimmune Encephalomyelitis: Recent Insights and Future Directions

**DOI:** 10.1007/s12035-021-02495-7

**Published:** 2021-09-03

**Authors:** Yan Mi, Jinming Han, Jie Zhu, Tao Jin

**Affiliations:** 1 Department of Neurology and Neuroscience Center, The First Hospital of Jilin University, Xinmin Street 71#, Changchun, 130021 China; 2 Present Address: Department of Neurology, Xuanwu Hospital, Capital Medical University, Beijing, China; 3 Department of Neurobiology, Care Sciences and Society, Karolinska Institutet, Karolinska University Hospital, Solna, Stockholm, Sweden

**Keywords:** PD-1, PD-L1, Multiple sclerosis, Experimental autoimmune encephalomyelitis, Immune tolerance

## Abstract

Multiple sclerosis (MS) is an autoimmunity-related chronic demyelination disease of the central nervous system (CNS), causing young disability. Currently, highly specific immunotherapies for MS are still lacking. Programmed cell death 1 (PD-1) is an immunosuppressive co-stimulatory molecule, which is expressed on activated T lymphocytes, B lymphocytes, natural killer cells, and other immune cells. PD-L1, the ligand of PD-1, is expressed on T lymphocytes, B lymphocytes, dendritic cells, and macrophages. PD-1/PD-L1 delivers negative regulatory signals to immune cells, maintaining immune tolerance and inhibiting autoimmunity. This review comprehensively summarizes current insights into the role of PD-1/PD-L1 signaling in MS and its animal model experimental autoimmune encephalomyelitis (EAE). The potentiality of PD-1/PD-L1 as biomarkers or therapeutic targets for MS will also be discussed.

## Introduction

Multiple sclerosis (MS) is a demyelinating disease of the central nervous system (CNS), attacking myelinated axons and leading to progressive physical disability. Genetic predisposition, epigenetic factors, lifestyle, and environmental factors all contribute to the risk of MS [1]. Previous studies indicated the immune dysfunction playing a primary role in the pathogenesis of MS [2–4]. Various types of immune cells including T cells, B cells, natural killer (NK) cells, dendritic cells (DCs), and macrophages/microglia are involved in the disease course [5]. Therefore, the modulation of immune response has drawn great attention recently. Several disease-modifying therapies (DMTs) have been discovered and approved to treat MS by targeting the immune system [6]. Non-specific therapeutic approaches may cause serious adverse events, such as leukemia and leukopenia [7]. Although the clinical development of DMTs has made encouraging success, there is still a remaining unmet need of highly specific treatment for MS [8].

Over the past years, a growing body of studies have suggested the crucial role of programmed cell death 1 (PD-1) and its ligand PD-L1 in maintaining immune tolerance and preventing autoimmunity. The involvement of PD-1/PD-L1 in MS has aroused increasing attention. In this review we first outline the cell-based immunomodulation of PD-1/PD-L1 signaling pathway. We then discuss current insight into the role of PD-1/PD-L1 in MS and its animal model experimental autoimmune encephalomyelitis (EAE) and summarize the regulation of PD-1/PD-L1 expression. In addition, the potentiality of PD-1/PD-L1 as biomarkers or therapeutic targets for MS and future directions of research will be introduced.

## PD-1 Receptor and Its Ligands

PD-1 (encoded by *Pdcd1* gene), also known as CD279, is an immunosuppressive co-receptor belonging to the CD28 family, mainly expressed on T lymphocytes, B lymphocytes and other immune cells [9]. The extracellular region of PD-1 is a single immunoglobulin variable-like domain and its cytoplasmic region contains an immunoreceptor tyrosine-based inhibitory motif (ITIM) and an immunoreceptor tyrosine-based switch motif (ITSM) [10]. Upon T-cell receptor (TCR) stimulation, the tyrosine residues of ITIM and ITSM are phosphorylated, recruiting src homology 2-domain-containing tyrosine phosphatase 1 (SHP1) and SHP2. Subsequently, downstream signals like CD3ζ, Zeta-chain-associated protein kinase 70 (ZAP-70) and protein kinase C-θ (PKC-θ) are dephosphorylated, resulting in the inhibition of TCR-mediated responses [11]. As for B cells, PD-1 mediates the dephosphorylation of Igβ, Syk, phospholipase C-γ2 (PLC-γ2) and extracellular signal-regulated kinase (ERK). These effects are dependent on the recruitment of SHP2 to the ITSM tyrosine [12].

PD-L1 (CD274, B7-H1) and PD-L2 (CD273, B7-DC) are the ligands of PD-1, belonging to the B7 family [13]. The two ligands share 40% identical amino acids [14]. The binding of PD-L2/PD-1 exhibits 2–six fold higher affinity than PD-L1/PD-1 interactions [15]. Although the binding affinity of PD-L2/PD-1 is high, relatively low expression of PD-L2 causes the interactions of PD-L1/PD-1 more competitive than PD-L2/PD-1. These diverse properties of PD-L1 and PD-L2 may contribute to the discrepancy of their involvement in PD-1 signal. PD-L1 is expressed on T cells and antigen-presenting cells (APCs) including B cells, DCs, monocytes and macrophages. PD-L1 can also be expressed on parenchymal tissues including vascular endothelial cells and pancreatic islet cells [16]. In contrast, PD-L2 is expressed on a few types of non-lymphoid cells, DCs and monocytes [17]. Different expression patterns of two ligands suggest a more important role of PD-L1 for tissue tolerance. The two ligands exhibited different functional features since PD-L1 is slightly more effective than PD-L2 on inhibiting the activation of T cells [18]. In support of this, a recent study demonstrated that the immunosuppressive role of PD-L2 in anti-tumor immunity is less significant than that of PD-L1 [19]. The role of PD-L1 and PD-L2 in modulating invariant natural killer T (iNKT)-cell-mediated airway hyperreactivity (AHR) in allergic asthma is unexpectedly opposite, which may be caused by distinct cytokine production [20]. In the graft versus host disease (GvHD)-like model, PD-1 was involved in the proliferation of alloreactive T cells via PD-1/PD-L2 pathway [21]. In some types of autoimmune models, PD-1/PD-L1, but not PD-1/PD-L2 interactions, has a crucial role in regulating T-cell functions, affecting the severity of the diseases [22–24]. However, some studies demonstrated that both PD-L1 and PD-L2 have the capacity for limiting autoimmunity [25, 26]. PD-L2 was able to exert its function through a receptor other than PD-1 [27]. We are just beginning to understand functional differences between PD-L1 and PD-L2. Owing to the growing insights into the role of PD-1/PD-L1 interactions in immune tolerance and translational therapies for autoimmunity, we put an emphasis on the immune modulation of PD-1/PD-L1 in this review.

## Application of PD-1/PD-L1 as an Immunotherapy for Cancer and Autoimmune Diseases

The expression of PD-L1 can be significantly upregulated on many malignant cell types, which is capable of constraining anti-tumor T-cell responses [28, 29]. Taken together with the discovery that PD-1 signaling induces T-cell dysfunction, PD-1/PD-L1 axis has been considered a promising strategy for breaking the tumor escape [30]. Both preclinical and clinical studies proved that blockade of PD-1 or PD-L1 inhibits tumor growth or delay progression in a broad spectrum of tumor types including solid and hematologic malignancies [31–35]. The Food and Drug Administration (FDA) approved anti-PD-1 antibody nivolumab as the first PD-1-targeting immune checkpoint blockade therapy for melanoma in 2014 [36]. In 2016, atezolizumab became the first FDA-approval PD-L1 inhibitor for treating urothelial carcinoma [36]. Currently, more than 5 PD-1/PD-L1 blockade therapies have been approved for the treatment of tumors [30]. Due to the complexity of immunomodulatory network and the heterogeneity of neoplasms and hosts, combinatorial regimens with PD-1/PD-L1 pathway blockade are just unfolding. PD-L1 expression has been demonstrated to be correlated to clinical response after PD-1-based therapy [37, 38]. Making use of potential predictive biomarkers is instructive to guide the rational application of the immune checkpoint blockade.

PD-1/PD-L1 not only occupies an important position in cancer immunotherapy, but also attracts much attention in the field of autoimmunity. Two decades ago, the deficiency of PD-1 was observed to cause lupus-like IgG3 deposition glomerulonephritis and destructive arthritis in mice [39]. Plenty of studies confirmed the engagement of PD-1 in the pathogenesis of autoimmune diseases [40–42]. The immune regulation of PD-1 is strain-specific [9]. For example, gene polymorphisms in *Pdcd1* are associated with the susceptibility of autoimmune diseases including rheumatoid arthritis (RA), type 1 diabetes mellitus (T1DM), systemic lupus erythematosus (SLE), ankylosing spondylitis (AS) and MS [43–47]. Similarly, PD-L1 deletion can promote autoimmunity [23, 48]. Certain autoimmune diseases may occur in cancer patients who are treated with anti-PD-1 or anti-PD-L1 antibodies [49, 50]. All these facts hint therapeutic potential to upregulate the PD-1/PD-L1 pathway for autoimmune diseases. Transgenic nonobese diabetic (NOD) mice over-expressing PD-L1 on islet cells significantly decreased the incidence of both spontaneous and lymphocyte transfer-mediated diabetes [51]. However, another study inferred an opposite conclusion that transgenic PD-L1 expression on the pancreatic islets promoted T cell-dependent spontaneous autoimmune diabetes and transplant rejection [52]. Her et al. detected the plasma concentrations of soluble PD-L1 in SLE, RA patients and healthy controls. Their results demonstrated that there were no significant differences among groups [53]. While the synovium and synovial fluid of RA was abundant in PD-1^+^ T cells and PD-L1^+^ macrophages [54], the expression of PD-1 on T cells had a positive correlation with the disease activity [55]. We propose that elevated expression of PD-1 may serve as a negative feedback on the breakdown of peripheral immune tolerance. Encouragingly, PD-1 activation induced by PD-L1-Fc fusion protein in vitro suppressed T-cell proliferation and decreased the production of interferon gamma (IFN-γ) from T cells in RA patients. Soluble PD-L1Ig treatment ameliorated the severity of collagen-induced arthritis (CIA) mice [54]. Stimulated PD-1 with PD-L1Ig fusion protein in experimental autoimmune glomerulonephritis (EAG) showed a marked reduction in corresponding parameters including albuminuria, serum creatinine, serum urea, segmental necrosis and tubular damage [56]. PD-L1Ig treatment resulted in low autoantibody production, delayed disease progression and prolonged survival in SLE murine model [57]. Experimental autoimmune neuritis (EAN) is an animal model of Guillain-Barré syndrome (GBS) with reactive T cells and macrophages accumulating in the peripheral nervous system. Intraperitoneal administration of PD-L1 attenuated disease severity by inhibiting inflammatory infiltration, demyelination and deficits of peripheral nerves in both preventative and therapeutic groups of EAN rats [58]. In summary, PD-1/PD-L1 may serve as a therapeutic target for autoimmune diseases.

## Association Between PD-1/PD-L1 and Immune Cells in MS/EAE

MS can be classified into relapsing–remitting MS (RRMS), primary progressive MS (PPMS), and secondary progressive MS (SPMS) [59]. The pathological hallmark of MS is focal demyelinating lesions in the CNS, including breakdown of the blood–brain barrier (BBB), immune cell infiltration, demyelination, oligodendrocyte loss, gliosis, axonal or neuronal degeneration [60]. Despite complex underlying mechanisms of MS remain incompletely understood, it is generally accepted that MS is triggered by autoimmune response toward CNS self-antigens. Immune dysregulation is thought to be crucial for the occurrence and development of MS. Inflammation is evident during different disease stages of MS, involving both innate and adaptive immune-mediated mechanisms. During acute phase DCs, macrophages, T cells and B cells from the periphery infiltrate across the BBB. They produce proinflammatory cytokines, chemokines and molecules to impair the myelin sheath. By contrast, during chronic phase diffuse inflammatory cells are infiltrated and CNS-resident microglia are chronically activated, resulting in constantly axonal injury, neuron loss, and pronounced atrophy of the gray and white matter [61]. Ultimately, neuroinflammation can be decreased and confined to the CNS compartments during disease progression. Therefore, peripheral immune cells and CNS-resident innate immune cells are key contributors of MS pathology at early and progressive stages, respectively. The former is the main target of current DMTs (summarized in Table 1).

**Table 1:** Currently FDA-approved disease modifying therapies for MS

DMTs	Route	Approved indication (year)	Mechanisms of action	Adverse effects
Interferon β	Injectable	CIS, RRMS (1993)	Regulates cytokine production of T cells; modulates B-cell trafficking across the BBB; increases CD56^bright^ NK cells	Injection site reactions; leukopenia; influenza-type symptoms; LFTs abnormalities; depression; headache
Glatiramer acetate	Injectable	RRMS (1996)	Competes for MHC binding of APCs; Inhibits Th1, Th17 cells and induce Th2, Treg cells; promotes neurotrophic factors secretion and remyelination	Local-injection-site reactions (tenderness, pruritus, erythema, or induration); rare systemic post injection reactions such as palpitations, chest pain, diaphoresis, and shortness of breath
Fingolimod	Oral	CIS, relapsing MS (2010)	Functional antagonist of receptors S1P1, S1P3 and S1P5, which prevents lymphocytes from exiting lymph nodes and blocks lymphocytes invasion into the CNS	Increased risk of macular edema and skin cancer; lymphopenia; bradycardia and atrioventricular conduction block on the first dose; opportunistic infections such as PML; LFTs abnormalities
Teriflunomide	Oral	CIS, RRMS (2012)	Dihydro-oratate dehydrogenase inhibitor, which inhibits pyrimidine synthesis, resulting in reduced lymphocytes proliferation	Hair thinning; LFTs abnormalities; lymphopenia increased blood pressure; nausea; diarrhea; teratogenicity
Dimethyl fumarate	Oral	CIS, relapsing MS (2013)	Inhibits invasion of neutrophils into the CNS; increases Treg cells, Th2 cells and CD56^bright^ NK cells; reduces Th1 cells, Th17 cells, CD8^+^ T cells and B cells; reduces oxidative stress	Flushing; diarrhea; nausea; upper abdominal pain; vomiting; LFTs abnormalities; lymphopenia; elevated risk of PML
Cladribine	Oral	RRMS, active SPMS (2019)	Synthetic deoxyadenosine analogue, which inhibits proliferation of T cells and B cells	Lymphopenia; infections; headache; teratogenicity
Siponimod	Oral	CIS, RRMS, active SPMS (2019)	Selective antagonist of receptors S1P1 and S1P5, inhibiting efflux of lymphocytes from lymph nodes	LFTs abnormalities; bradycardia; macular edema; hypertension; convulsions
Mitoxantrone	Intravenous	Aggressive RRMS, SPMS, PRMS (2006)	Topoisomerase inhibitor, which decreases T cells and B cells; inhibits antigen presentation, macrophages proliferation and pro-inflammatory cytokines secretion	Systolic cardiac dysfunction; acute myeloid leukemia; nausea; anemia; amenorrhea; alopecia; colon cancer
Natalizumab	Intravenous	RRMS (2006)	A monoclonal antibody that binds α4β1-integrin, inhibiting migration of inflammatory cells across the BBB; increases CD56bright NK cells	Allergic reaction; opportunistic infections such as PML; headache; fatigue; joint pain; chest discomfort
Alemtuzumab	Intravenous	RRMS (2014)	A monoclonal anti-CD52 antibody which depletes both T cells and B cells; increases Treg cells and CD56^bright^ NK cells	Increased risk of autoimmune diseases, such as autoimmune thyroiditis, idiopathic thrombocytopenic purpura
Ocrelizumab	Intravenous	RRMS, PPMS (2017)	A monoclonal anti-CD20 antibody, leading to cells lysis of B cells; increases Breg cells	Infusion reactions; opportunistic infections

*Abbreviations*: *FDA*, Food and Drug Administration; *DMTs*, disease modifying therapies; *CIS*, clinically isolated syndrome; *RRMS*, relapsing–remitting MS; *SPMS*, secondary progressive MS; *PRMS*, progressive relapsing MS; *PPMS*, primary progressive MS; *BBB*, blood–brain barrier; *MHC*, major histocompatibility; *APCs*, antigen-presenting cells; *CNS*, central nervous system; *NK*, natural killer; *S1P*, sphingosine 1-phosphate; *LFTs*, liver function tests; *PML*, progressive multifocal leukoencephalopathy

Taken together, T cells, B cells, NK cells, DCs, and microglia/macrophages are actively involved in the pathogenesis of MS. Detailed roles of each immune cell in MS pathogenesis are complicated, which may depend on the disease stage and microenvironment. Precise functional regulation of these immune cells is thus of uttermost importance in potential therapeutic development for MS. A growing number of evidence has shown that PD-1/PD-L1 signaling exerts complex regulatory impacts on immune responses. Here we discuss the moderating role and potential mechanisms of PD-1/PD-L1 signaling in various immune cells during MS/EAE.

### T Lymphocytes

T lymphocytes are critical in adaptive immune responses, modifying the balance between protective immunity and tolerance. The autoimmunity of EAE is mainly mediated by effector T cells, suggesting a key role of antigen-specific T cells in MS/EAE. Specifically, Th1, Th17 cells and CD8^+^ T cells are pathogenic in MS/EAE, while regulatory T cells (Tregs) and Th2 cells suppress autoimmune responses [61]. In MS/EAE, central and peripheral tolerance of T cells can be broken through defective functions of Tregs and/or impaired immunosuppressive modulation of effector T cells. The autoreactive T cells that target CNS antigens can be activated in the periphery and then differentiated into CD4^+^ Th1, Th17 cells, and CD8^+^ T cells. These effector T cells are infiltrated and re-activated in the CNS, causing neuroinflammation.

**Fig. 1:**
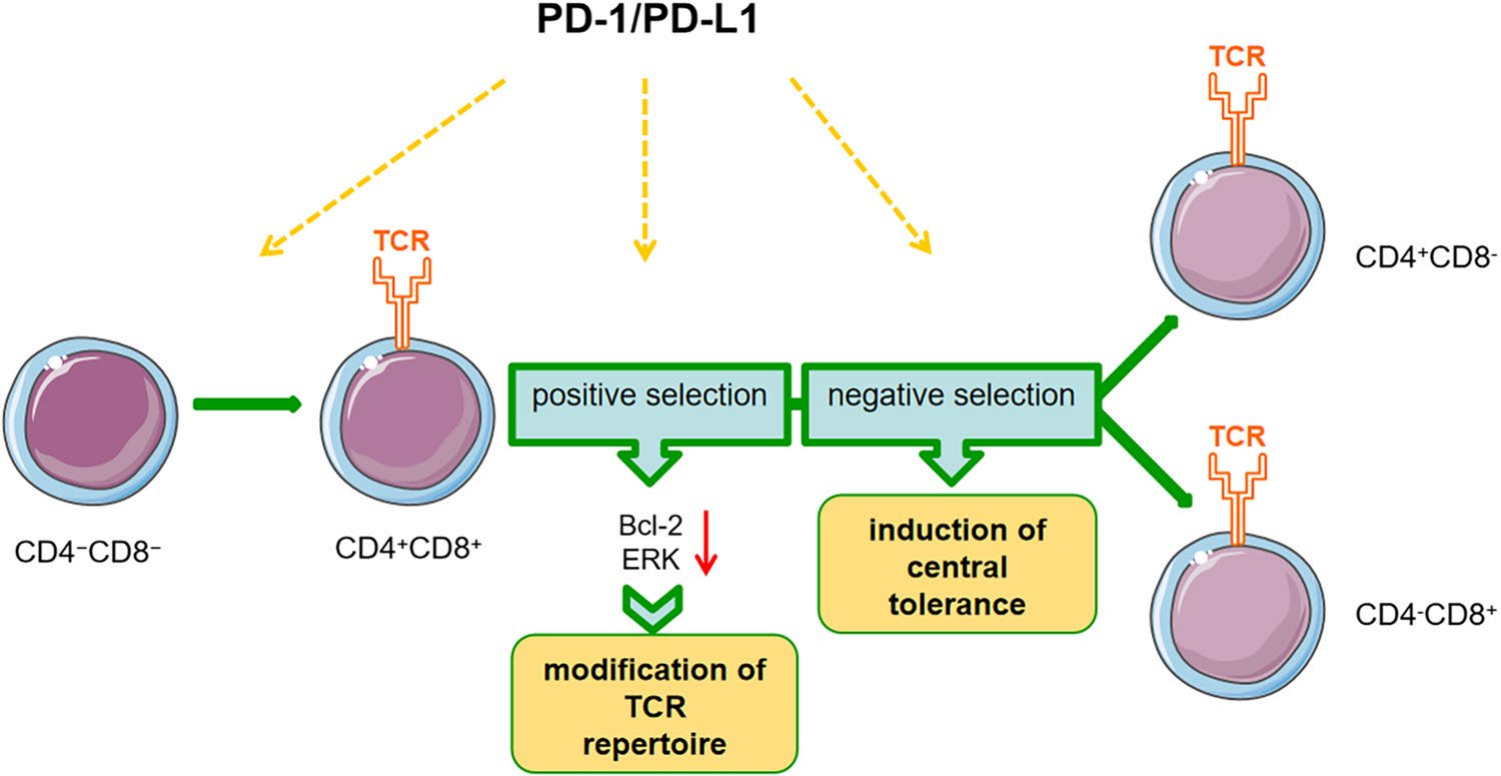
PD-1/PD-L1 regulates T cell development. PD-1 and PD-L1 are involved in T cell development in the thymus. PD-1 is expressed on CD4^−^CD8^−^ double-negative thymocytes and PD-L1 is widely expressed in the thymic cortex. PD-1 modulates TCR development and thymocyte transition to the CD4^+^CD8^+^ double positive cells. PD-1 signaling regulates the TCR repertoire by controlling TCR signaling thresholds during positive selection. Loss of PD-L1 signaling at this stage results in a high number of double positive T cells. TCR/PD-1 cocross-linking inhibits Bcl-2 upregulation and ERK phosphorylation. Both of them are downstream of TCR signaling and are essential for thymocyte development. PD-1/PD-L1 also plays a role in negative selection, contributing to the induction of central tolerance. *Abbreviations*: PD-1, programmed cell death 1; PD-L1, programmed cell death 1 ligand 1; TCR, T-cell receptor; Bcl-2, B-cell lymphoma-2; ERK, extracellular signal–regulated kinase

PD-1 and PD-L1 are involved in central tolerance mechanisms of T cells. The goal of central tolerance is to delete self-reactive clones during negative selection of T cell development, thus avoiding autoimmunity. PD-1/PD-L1 interactions contribute to T cell development (Fig. 1). The expression of PD-1 can be noted in CD4^−^CD8^−^ double-negative (DN) thymocytes, and PD-L1 is widely expressed in the thymic cortex. PD-1 deficiency promoted thymocyte transition from DN to CD4^+^CD8^+^ double positive (DP) thymocytes and decreased the efficiency of positive selection [62]. PD-1 may regulate the TCR repertoire of mature T lymphocytes by controlling TCR signaling thresholds. In addition, transgenic mice that constitutively overexpressed PD-1 on CD4^+^CD8^+^ thymocytes displayed defects in positive selection and thymocyte maturation, which can be resolved upon the elimination of PD-L1 [63]. PD-L1-deficient mice had great numbers of DP and CD4^+^ thymocytes, indicating that PD-L1 is also involved in regular thymic selection. Further mechanistic experiments showed that TCR/PD-1 cocross-linking inhibited B-cell lymphoma-2 (Bcl-2) upregulation and extracellular signal-regulated kinase (ERK) phosphorylation, both of which are downstream of TCR signaling playing a role in thymocyte development. Moreover, *Pdcd1* contributes to the modulation of negative selection at the DP stage and has been identified as a candidate gene of defective central tolerance in NOD mice [64, 65]. So far, how PD-1 and PD-L1 regulate thymic selection and modulate TCR signaling thresholds are poorly defined.

**Fig. 2:**
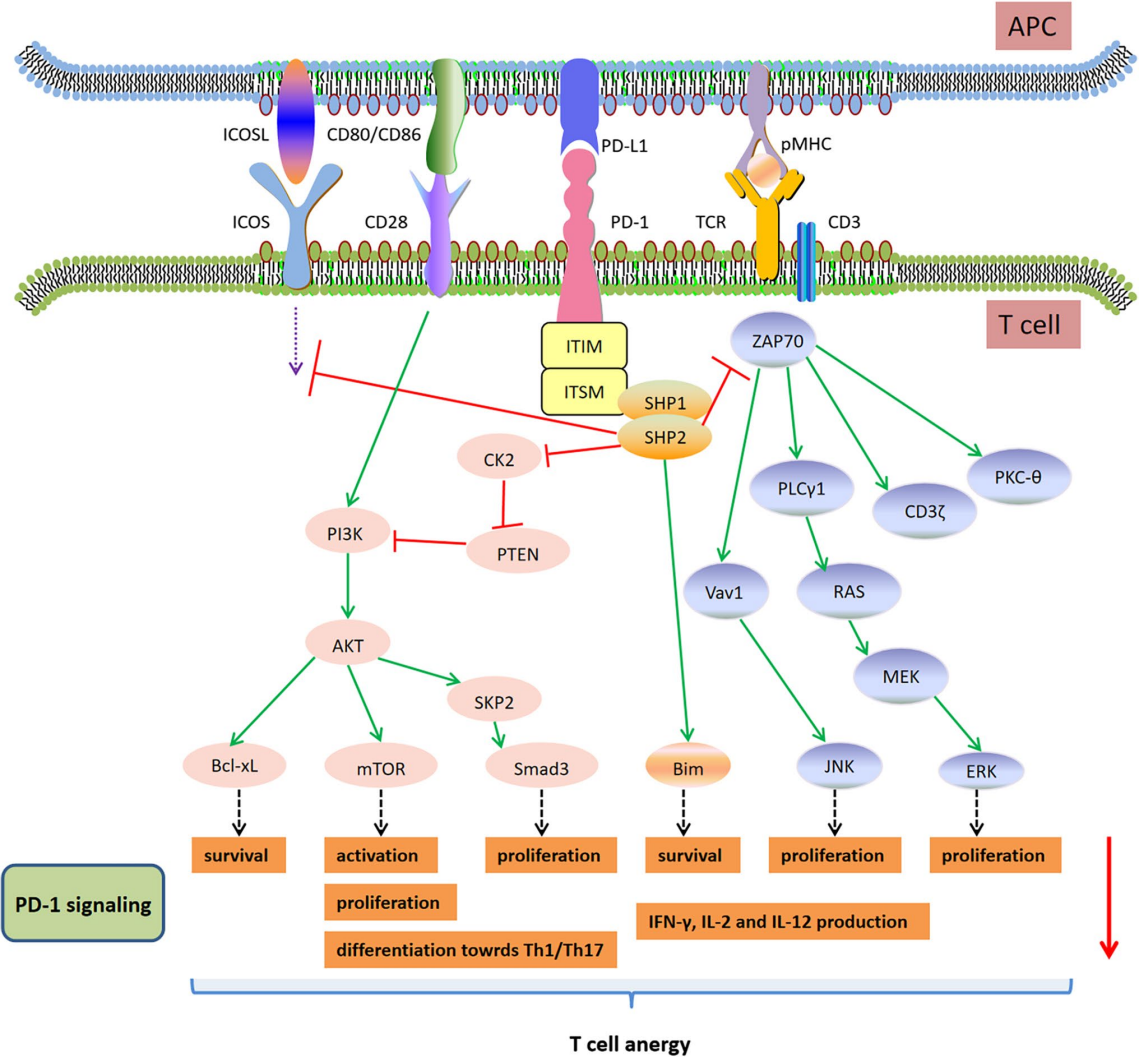
PD-1 signaling enfeebles T cell functions. When engaged with PD-L1, PD-1 transmits a co-inhibitory signal to hinder T cell activation through the phosphorylation of ITIM and ITSM, thereby recruiting the tyrosine phosphatase SHP1 and SHP2. PD-1 then mediates decreased phosphorylation of TCR signaling transducers including ZAP-70, PKC-θ, CD3ζ, Vav1, PLCγ1, and further downstream molecules JNK, RAS, MEK, and ERK. In addition to TCR signaling, PD-1 can also hamper co-stimulatory molecules CD28 and ICOS. Tyrosine phosphorylation of CD28 recruits and activates PI3K, resulting in AKT phosphorylation. PI3K-AKT activation facilitates enhanced glucose uptake, glycolysis and cellular metabolism of T cells, which can be restrained through PD-1 engagement. The inhibition of the PI3K-AKT pathway through PD-1 involves PTEN dephosphorylation, mediated by CK2 downregulation. PD-1 blocks cell cycle progression through the G1 phase by inhibiting SKP2 expression and Smad3 activation, which is mediated by PI3K-AKT, RAS and MEK-ERK pathways, leading to the downregulation of glycolytic enzyme activity and decreased T cell expansion. PD-1 signaling alters T cell differentiation by decreasing Th1 cells and Th17 cells as well as increasing Th2 cells and Tregs, which induced by the PI3K-AKT-mTOR pathway. During T cell activation, CD28 stimulation retains T cell survival by upregulating the expression of anti-apoptotic gene *Bcl-xL*. PD-1 ligation hampers Bcl-xL expression by limiting PI3K activation. PD-L1 can enhance the death of effector CD8^+^ T cells through the upregulation of pro-apoptotic molecule Bim, causing the depletion of memory CD8^+^ T cells. Meanwhile, PD-1 signaling alters cytokine production of T cells by attenuating proinflammatory cytokine (IFN-γ, IL-2 and IL-12) production and increasing anti-inflammatory cytokine IL-10 secretion. *Abbreviations*: PD-1, programmed cell death 1; PD-L1, programmed cell death 1 ligand 1; APC, antigen-presenting cell; TCR, T-cell receptor; ICOS, inducible T-cell co-stimulator protein; ICOSL, inducible T-cell co-stimulator ligand; ITIM, immunoreceptor tyrosine-based inhibitory motif; ITSM, immunoreceptor tyrosine-based switch motif; SHP, src homology 2-domain-containing tyrosine phosphatase; ZAP-70, Zeta-chain-associated protein kinase 70; PKC-θ, protein kinase C-θ; PLCγ1, phospholipase C gamma 1; JNK, c-Jun N-terminal kinase; RAS, retrovirus-associated DNA sequences; MEK, extracellular signal–regulated kinase kinase; ERK, extracellular signal–regulated kinase; PI3K, phosphatidylinositol 3-kinase; mTOR, mammalian target of rapamycin; PTEN, phosphatase and tensin homolog; CK2, casein kinase 2; SKP2, S-phase kinase-associated protein 2; Bcl-xL, B-cell lymphoma-extra large; Bim, Bcl-2 interacting mediator of cell death

T cell activation requires both antigen-specific signals from peptide-MHC complexes and antigen-independent signals from co-signaling molecules. Two sets of co-signaling molecules are cell-surface molecules that transduce signals into T cells to modulate TCR signaling positively (co-stimulatory molecules, such as CD28) or negatively (co-inhibitory molecules) [66]. As described before, PD-1 primarily transmit a co-inhibitory signal through the tyrosine phosphatase SHP1 and SHP2 to enfeeble T-cell activation when engaged with PD-L1 [11]. In other words, PD-1/PD-L1 paralyzes T cells in a hyporesponsive state called “anergy,” which is responsible for peripheral tolerance and immune homeostasis (Fig. 2). In the condition of chronic viral infection, T cell exhaustion is maintained in order to avoid severe disease, while the administration of PD-1/PD-L1 antibodies enhances T cell motility and restores T cell ability to lyse target cells and secrete proinflammatory cytokines such as IFN-γ, causing fatal diseases [67, 68]. In autoimmune diseases, the upregulation of PD-1 signaling on T cells promoted the resolution of inflammation and ameliorated disease severity by inducing the anergic state of T cells and controlling T cell responses [54, 69]. PD-1-mediated decreased phosphorylation of TCR signaling molecules including ZAP-70, PKC-θ, CD3ζ, Vav1, PLCγ1 and further downstream molecules including c-Jun N-terminal kinase (JNK), retrovirus-associated DNA sequences (RAS), extracellular signal-regulated kinase kinase (MEK) and ERK [11, 68, 70, 71]. Apart from TCR signaling, co-stimulatory molecules CD28 and inducible T-cell co-stimulator protein (ICOS) are also reported to be the target signalings of PD-1/PD-L1 [72] Tyrosine phosphorylation of CD28 recruits and activates phosphatidylinositol 3-kinase (PI3K), resulting in serine-threonine kinase AKT phosphorylation. PI3K-AKT activation facilitates enhanced glucose uptake, glycolysis and cellular metabolism of T cells, which can be restrained through PD-1 engagement [10]. It is implied that the inhibition of the PI3K-AKT pathway through PD-1 involves phosphatase and tensin homolog (PTEN) dephosphorylation, mediated by casein kinase 2 (CK2) downregulation [73]. A recent study demostrated that CD28 is a primary target of PD-1-mediated inhibition, which has yielded significant insight into the signaling pathways affected by PD-1 activation [74]. The fluorescence resonance energy transfer (FRET)-based assay indicated that SHP2, but not SHP1, is the major effector of PD-1. SHP2 recruitment requires Lck-mediated dual phosphorylation of PD-1. Notably, PD-1-SHP2 displays preferential dephosphorylation of CD28, rather than TCR. Nevertheless, data in vitro revealed that enhanced CD28 signal can overcome PD-1-induced inhibition by augmenting interleukin-2 (IL-2) production in the presence of anti-CD3/CD86, which implies relatively low susceptibility of CD28 signal to PD-1-mediated negative regulation [72, 75]. The contradictions of these findings may be attributed to complicated dependence of diverse T cell states on TCR and CD28 signals. Interestingly, SHP2 is dispensable for PD-1 signaling and the induction of T-cell exhaustion in vivo [76]. Therefore, there is a degree of redundancy in downstream signalings of PD-1. Specific effects of PD-1 on related intracellular signaling pathways in diverse T cell states (naïve, effector, memory, anergic or exhausted T cells) need further investigation.

In 2002, Laura L and colleagues firstly discovered that PD-L1/PD-1 interactions suppress CD3-mediated T cell proliferation by restricting the cells from entering into cellular division cycle, which related to an impaired production of autocrine growth factor IL-2 [77]. CD28 signal is involved in the modulation, as the induction of IL-2 upon CD28 engagement has been well documented. Another study pointed out that IL-2, IL-7 and IL-15 can overcome PD-1-mediated inhibition of T cell proliferative responses through transcriptional regulation of transcription 5 (STAT5) [72]. PD-1 blocks cell cycle progression through the G1 phase by inhibiting S-phase kinase-associated protein 2 (SKP2) and Smad3 activation, which is mediated by PI3K-AKT, RAS and MEK-ERK pathways, and leading to the downregulation of glycolytic enzyme activity and reduction of T cell expansion [71]. Decreased expression of PD-1 on peripheral blood T cells can enhance T cell proliferation, produce proinflammatory cytokines (IFN-γ, IL-2 and IL-12) and reduce anti-inflammatory cytokine secretion in human coronary artery disease [78].

Accumulating evidence suggests that PD-1/PD-L1 interactions play a role in the differentiation of activated T cells. PD-L1Ig treatment altered T cell differentiation by decreasing the percentages of Th1 and Th17 cells as well as increasing Th2 cells and Tregs in spleens of EAN rats [58]. Meanwhile, phosphorylated PI3K, AKT, and mTOR were suppressed, indicating a pivotal role of PI3K-AKT-mTOR axis in regulating T cell differentiation affected by PD-L1. Furthermore, the overexpression of PD-L1 induces Th1 cells converting into Tregs, shielding immune-deficient murine hosts from GvHD after transplantation [79]. This phenotype transformation of Th1 cells is associated with SHP1/2 recruitment and STAT1 inactivation, dependent on the PD-1/PD-L1 signaling [79].

Tregs are involved in peripheral tolerance modulation by inhibiting pathogenic effector T cell-mediated tissue damage [80]. Tregs are expressed the transcription factor forkhead box protein P3 (Foxp3), which divided into two subpopulations: naturally occurring (nTreg) and induced Treg (iTreg). PD-L1 is constitutively expressed by Tregs. Due to a key role of PD-1/PD-L1 and Tregs in the maintenance of peripheral tolerance, a large amount of studies have focused on the regulation of PD-1/PD-L1 on Treg differentiation and functions. When naïve CD4^+^ T cells were co-cultured with PD-L1^−/−^ APCs in the presence of anti-CD3 and transforming growth factor beta (TGF-β), decreased Foxp3^+^ Tregs were observed [81]. Besides, PD-L1Ig enhanced the maintainment of Foxp3 expression on iTregs and promoted suppressive efficiency of iTregs. In mechanistic experiments, the levels of AKT, mTOR and S6 ribosomal protein phosphorylation were significantly diminished when naïve T cells were cultured in the presence of PD-L1, since PD-L1 upregulated the expression of PTEN which antagonizes the PI3K-AKT pathway [81]. Another study reported the intrinsic function of PD-1 in maintaining Foxp3 stability by downregulating endo-lysosomal protease asparaginyl endopeptidase (AEP) in iTregs during experimental autoimmune colitis and GvHD [82]. Experimental autoimmune uveitis (EAU) is a mouse model of human autoimmune uveitis. Muhammad et al. recently demonstrated that a protective role of Treg in preventing the mice from EAU was PD-1-dependent [83]. Investigation of pregnancy in animal models also suggested an important role of PD-1/PD-L1 and Tregs interactions in fetomaternal tolerance. PD-L1 blockade led to decreased allogeneic fetal survival rates, associated with increased Th17 cells and a reduction of Tregs [84]. Importantly, mice with partial Foxp3 insufficiency developed early-onset lympho-proliferation and lethal autoimmune pancreatitis when PD-1 is deficient, which can be rescued by the transfer of PD-1-Foxp3^+^ Tregs [85]. Based on these studies, we conclude that PD-1/PD-L1 signaling preferentially pushes T cells toward an inhibitory Treg fate, while the regulation of PD-1/PD-L1 and Tregs is not completely overlapped regarding peripheral immune tolerance. PD-1-deficient Tregs were sufficient to rescue the autoimmune phenotype, indicating that PD-1 signaling reduces immunosuppressive function of Tregs [85]. Furthermore, PD-1 may also exert negative effects on Treg-mediated immunosuppression in tumor and chronic infection [86, 87]. Different immune status and local microenvironment may partially explain the discrepant findings regarding the effect of PD-1/PD-L1 on Treg functions.

In 1992, Honjo and colleagues identified the expression of PD-1 was strongly induced upon programmed cell death and thereby played a role in apoptosis [88]. They discovered that overexpression of PD-1 cDNA failed to induce apoptosis of T cells, suggesting that PD-1 may not trigger the apoptotic signaling directly [89]. Subsequent studies indicated that the engagement of PD-1/PD-L1 can indirectly hinder T cell survival by impacting the expression of apoptotic-related genes [10, 90]. During T cell activation, CD28 costimulation and TCR signaling retain T cell-survival by upregulating the expression of B-cell lymphoma-extra large (Bcl-xL), an anti-apoptotic gene. PD-1 ligation hampered Bcl-xL expression via limiting the PI3K activation, causing impaired T cell survival [10]. Specifically, PD-1 impacts the survival of anti-viral T cells in chronic infection. The expression of PD-1 on CD8^+^ T cells (also known as cytotoxic T lymphocytes, CTLs) augmented their sensitivity to both spontaneous and CD95/Fas-induced apoptosis [91]. Similarly, PD-L1 enhanced the death of effector CD8^+^ T cells through the upregulation of pro-apoptotic molecule Bcl-2 interacting mediator of cell death (Bim), promoting the depletion of memory CD8^+^ T cells accordingly [90]. However, Pulko et al. found that PD-L1 deficiency increased the apoptosis and the susceptibility of effector CD8^+^ T cells to Ca-dependent and Fas ligand-induced killing by other CTLs, and downregulated Bcl-xL expression [92]. With the contradictory results, more efforts are needed to gain a better understanding of this issue.

### B Lymphocytes

Immunoglobulin G oligoclonal bands (OCBs) were evident in cerebrospinal fluid (CSF) of most patients with MS and anti-CD20 therapies are increasingly used as DMTs in MS. B cells are believed to display a pathogenic role in MS.

**Fig. 3:**
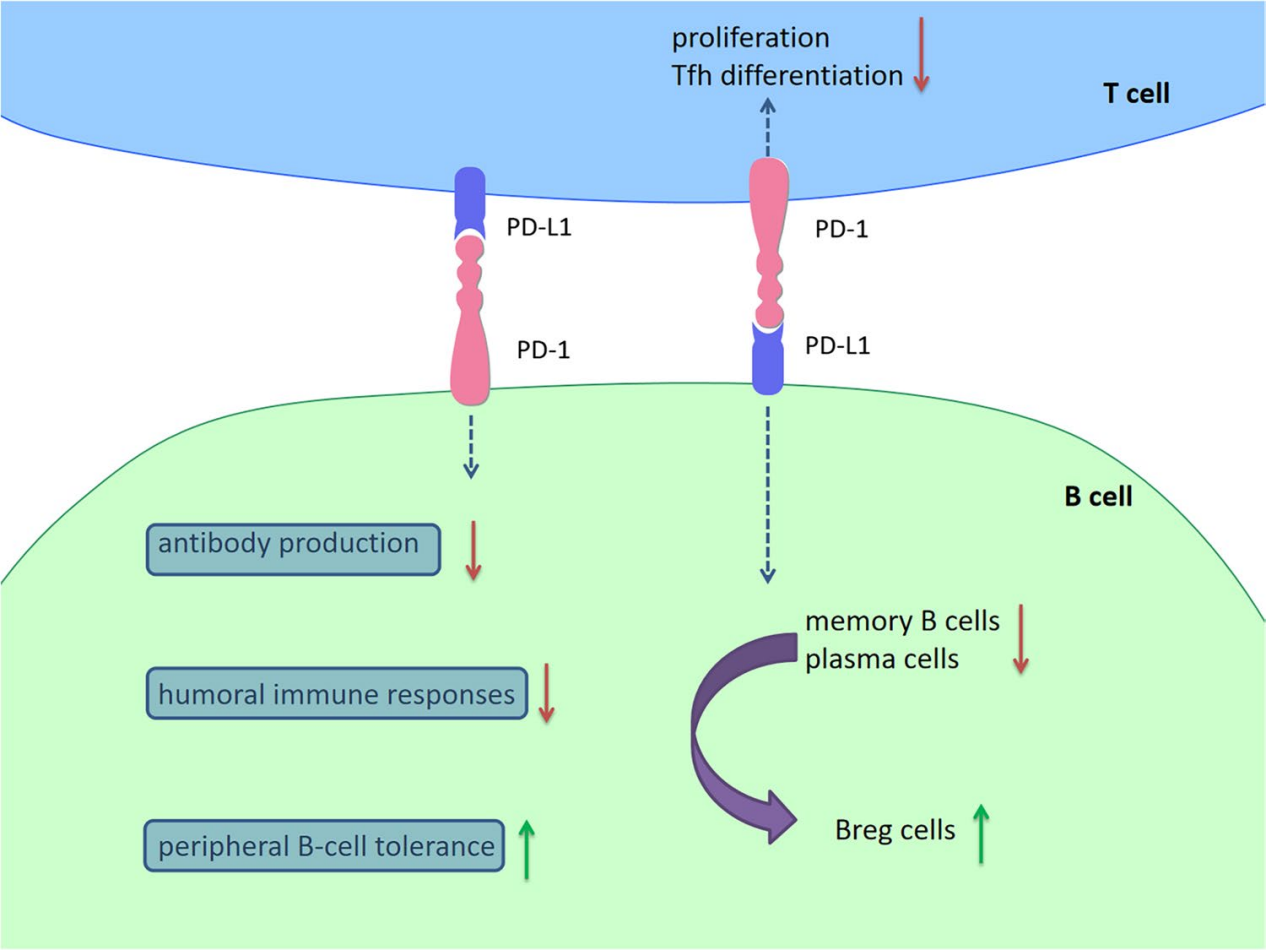
PD-1/PD-L1 signaling in B cells. PD-1 is expressed on many subpopulations of B cells. GC B cells and plasma cells also express PD-L1. PD-L1^−/−^ B cells stimulated a high proliferation of CD4^+^ T cell in vitro. With the expression of PD-L1, Tregs inhibited autoreactive B cells and induced peripheral B cell tolerance directly through PD-1 in vivo. Elevated expression of PD-L1 on Breg cells suppressed the expansion and differentiation of Tfh cells via alterations in downstream signaling pathways following PD-1 ligation. The reduction of Tfh cells limited B-cell fate by limiting both memory B cell development and terminal differentiation to plasma cells, which dramatically inhibited antibody production and subsequent humoral responses. Blockade of PD-L1 can enhance humoral immunity by upregulating the generation of Tfh cells, and blockade of PD-1 can enhance antigen-specific immunoglobulin production. *Abbreviations*: PD-1, programmed cell death 1; PD-L1, programmed cell death 1 ligand 1; GC, germinal center; Breg cells, regulatory B cells

PD-1 is expressed on many subpopulations of B cells. Germinal center (GC) B cells and plasma cells express PD-L1 [93–95]. Similar to T cells, a negative effect of PD-1 signaling on B cell function is strongly suggested. PD-1-knockout mice developed high levels of auto-antibodies [96]. PD-L1^−/−^ B cells stimulated a high proliferation of CD4^+^ T cells in vitro [48]. With the expression of PD-L1, Tregs inhibited autoreactive B cells and induced peripheral B cell tolerance directly through PD-1 in vivo [97]. Cellular mechanisms include recruiting SHP2 to its phosphotyrosine and dephosphorylating key signal transducers of BCR signaling [12]. Elevated expression of PD-L1 on regulatory B (Breg) cells suppressed follicular helper T (Tfh)-cell expansion and differentiation via alterations in downstream signaling pathways following PD-1 ligation [98]. The reduction of Tfh cells limited B-cell fate by limiting both memory B cell development and terminal differentiation to plasma cells, which dramatically inhibited antibody production and subsequent humoral responses. Consistent with this, blockade of PD-L1 can enhance humoral immunity by upregulating the generation of Tfh cells [99], and blockade of PD-1 can enhance antigen-specific immunoglobulin production [100]. However, PD-1 has also been reported to be essential for GC responses, including GC B cell survival, the formation and affinity of long-live plasma cells, optimal GC localization and activity of Tfh cells [101, 102]. It is worth mentioning that the expression of PD-L1 is increased, while PD-1 expression is decreased in GC B cells and PD-1 is upregulated in Tfh cells [95, 101]. One reasonable explanation is that GC B cells downregulate PD-1 to reduce the interactions between B cells and Tfh cells by PD-L1-PD-1 ligation. In addition, PD-1/PD-L1 interactions between Tfh cells and B cells dampen TCR signaling and reduce the ligand sensitivity of Tfh cells, thereby enforcing a stringent selection threshold for competing B cells to promote affinity maturation [102] (Fig. 3).

### Natural Killer Cells

**Fig. 4:**
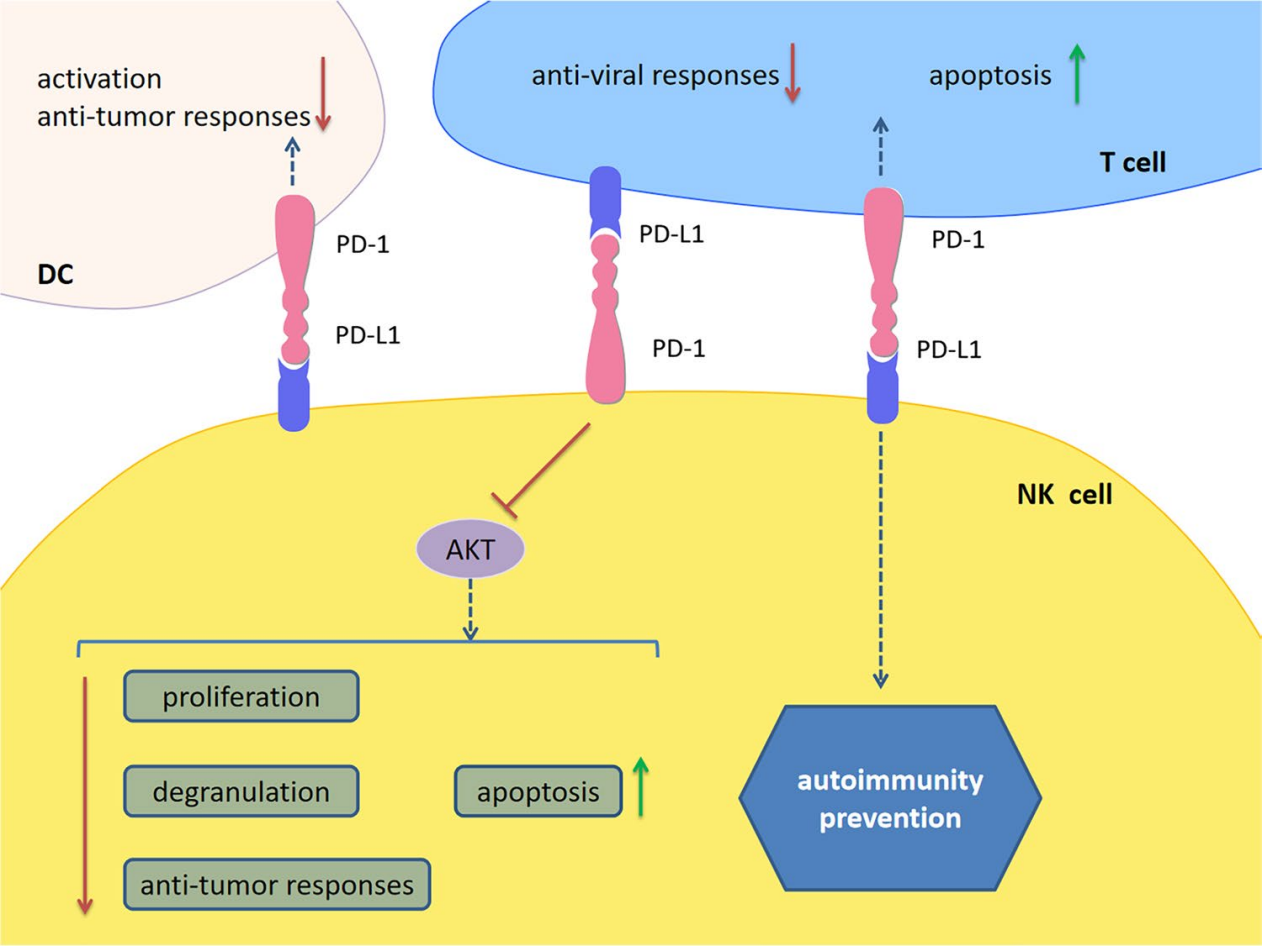
PD-1/PD-L1 signaling in NK cells. PD-1^+^ NK cells display a reduced proliferative capability in response to cytokines, low degranulation, impaired anti-tumor activity and increased apoptosis that can be partially restored by PD-1/PD-L1 blockade. PD-1 exerts its inhibitory effects on NK cells through interfering with AKT phosphorylation. PD-L1 on NK cells can interact with PD-1 on DCs and then inhibit DCs activation, exerting negative impacts on anti-tumor immunity. During acute and chronic viral infection, liver-resident NK cells suppress the anti-viral responses of hepatic T cells via PD-1/PD-L1 interactions. Besides, NK cells which highly expressed PD-L1 play an immunosuppressive role in autoimmunity. *Abbreviations*: PD-1, programmed cell death 1; PD-L1, programmed cell death 1 ligand 1; NK cells: natural killer cells; DCs, dendritic cells

NK cells act as sentinels for detecting aberrant cells. Unlike T and B cells, NK cells mediate immune defense without prior antigen sensitization. They discriminate target cells such as infected or malignant cells through a molecular detection system including a variety of cell surface activating and inhibitory receptors. When activated, NK cells target cell killing through release of perforin- and granzyme-containing cytotoxic granules, which is accompanied by secretion of proinflammatory and immunoregulatory cytokines [103]. NK cells are significant players in MS/EAE. NK cell depletion before immunization diminished the onset and severity of EAE, along with decreased lymphocytes and DCs infiltration into the CNS (104). However, two main subsets of human NK cells have different effects on MS. NK cell subsets are increased in the CSF of MS patients, and the regulatory/effector (CD56^bright^CD16^−^/CD56^dim^CD16^+^) NK ratio is also increased remarkably [105]. Many current DMTs can increase the CD56^bright^ regulatory NK cell population in peripheral blood, which has the capability to suppress autologous CD4^+^ T cell proliferation through direct cytotoxicity [106, 107]. PD-1 is highly expressed on a small percentage of human peripheral blood NK cells in one quarter of healthy individuals, and a potential correlation is established between human cytomegalovirus (HCMV) seropositivity and the presence of PD-1^+^ NK cells. Latent chronic diseases such as viral infection may contribute to the induction and expression of PD-1 on NK cells (108). PD-1^+^ NK cells display a reduced proliferative capability in response to cytokines, low degranulation, impaired anti-tumor activity and increased apoptosis that can be partially restored by PD-1/PD-L1 blockade [108, 109]. Similar to T cells, PD-1 exerted its inhibitory effect on NK cells through interfering with AKT phosphorylation [109]. PD-L1 on NK cells can interact with PD-1 on DCs and inhibit the activation of DCs, exerting negative impacts on anti-tumor immunity [110]. During acute and chronic viral infection, liver-resident NK cells suppress the anti-viral responses of hepatic T cells via PD-1/PD-L1 interactions [111]. Besides, NK cells which highly expressed PD-L1 play an immunosuppressive role in autoimmunity. PD-L1 expression on NK cells was upregulated when stimulated by IL-18. Adoptive transfer of these cells into streptozotocin-treated mice led to a delayed diabetes development and partial disease prevention with the mechanism involving apoptosis induction of activated antigen-specific CD8^+^ T cells [112]. However, a study of enteric microbial infection in mice suggested that increased PD-1 expression can boost the function of NK cells and promote protective immunity by increasing the expression and production of granzyme B and perforin of mucosal NK cells (113) (Fig. 4).

### Dendritic Cells

DCs play both immunogenic and immunoregulatory roles in MS/EAE. Upon pathological stimulation, mature DCs activate naïve T cells in the periphery and promote them to differentiate into effector cells, resulting in the release of proinflammatory cytokines. Activated T cells can be re-activated upon encounter with CNS-resident DCs which present myelin-derived epitopes [111]. Tolerogenic DCs are a steady state of immature, maturation-resistant DCs that express low levels of co-stimulatory molecules (CD80, CD86, and CD40), high levels of co-inhibitory molecules (PD-L1 and CD95L) and have an ability to induce Tregs instead of Th1/Th17 responses [112]. Tolerogenic DCs induce stable antigen-specific immunological hyporesponsiveness in myelin-reactive T cells from RRMS patients [114]. Administration of tolerogenic DCs decreased the incidence and severity of EAE through the induction of Tregs, the reduction of Th1 and Th17 cells, and the production of IL-10 (115, 116).

**Fig. 5:**
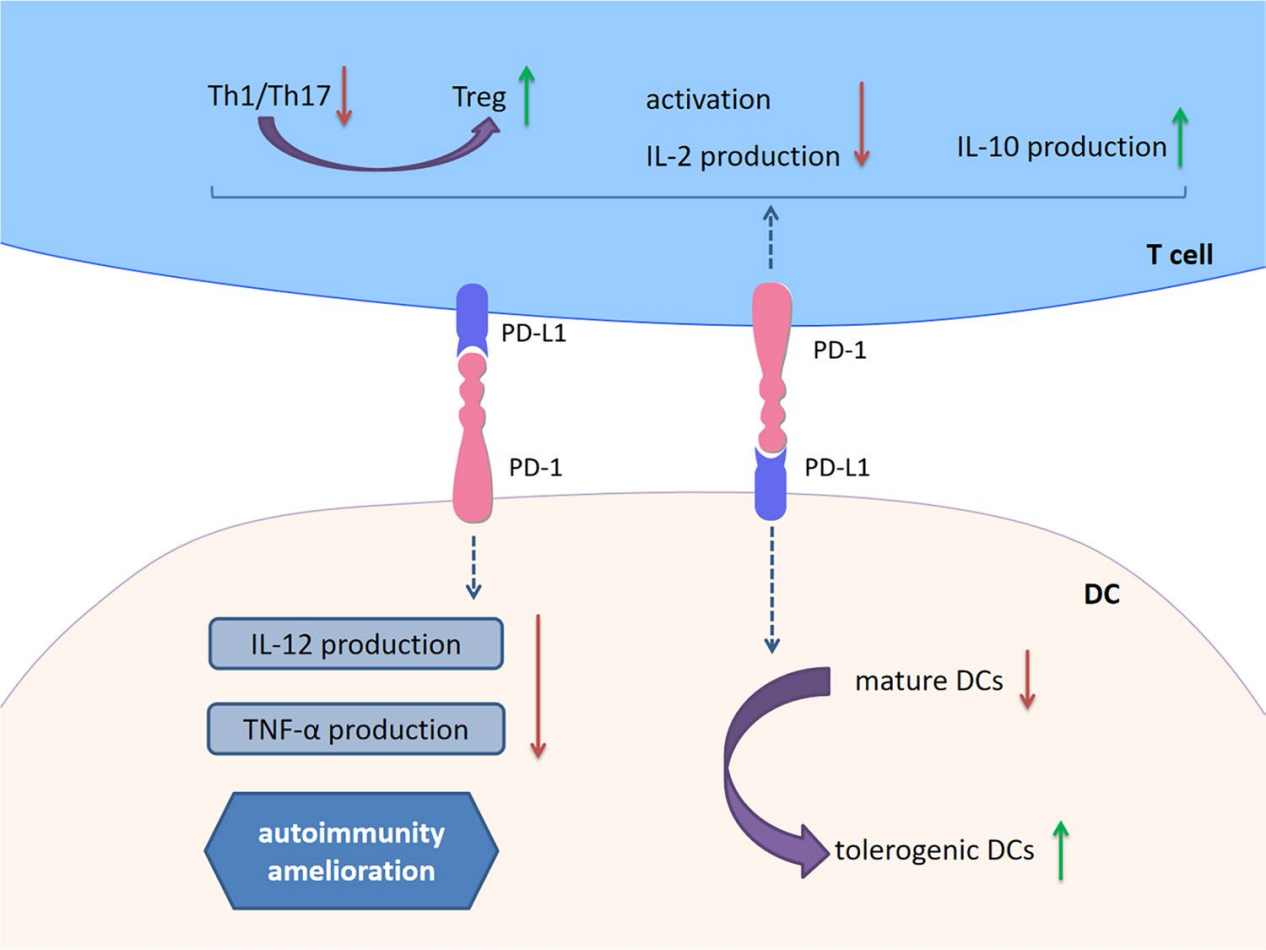
PD-1/PD-L1 signaling in DCs. Overexpressed PD-L1 in DCs may impair CD4^+^ T cell activation and IL-2 production in vitro. Tolerogenic DCs that highly express PD-L1 enhance the expression of PD-1 in T cells both in vivo and in vitro. Soluble PD-1 can induce a tolerogenic DC phenotype via reversing signaling by PD-L1 into DCs. Due to the powerful role in immune tolerance induction, tolerogenic DC has been considered a therapeutic target for autoimmune diseases. Besides, PD-1 expression on DCs can negatively modulate DC functions and impede immune responses by interfering the production of DC-derived IL-12 and TNF-α. *Abbreviations*: PD-1, programmed cell death 1; PD-L1, programmed cell death 1 ligand 1; DCs, dendritic cells; IL, interleukin; TNF-α, tumor necrosis factor alpha

Overexpressed PD-L1 in DCs may impair CD4^+^ T cell-activation and IL-2 production in vitro [117]. Also, tolerogenic DCs enhance the expression of PD-1 in T cells both in vivo and in vitro [118, 119]. Interestingly, soluble PD-1 can induce a tolerogenic DC phenotype via reversing signaling by PD-L1 into DCs [117]. Due to the powerful role in immune tolerance induction, tolerogenic DCs that highly express PD-L1 have been considered a therapeutic target for GvHD and autoimmune diseases [118, 120]. Besides, PD-1 expression on DCs can negatively modulate DC functions and impede immune responses by interfering the production of DC-derived IL-12 and tumor necrosis factor alpha (TNF-α) [121]. It is inferred that PD-L1-expressing cells may have the potential to suppress T cell responses by directly engaging PD-1 on DCs and inhibiting the function of DCs. DC-DC interplay through PD-L1/PD-1 may also play a role in immune modulation (Fig. 5).

### Macrophages/Microglia

Microglia are tissue macrophages in the CNS, playing a critical role for neural development, synaptic pruning and CNS homeostasis [122]. In MS/EAE, microglia are viewed as a “double edged sword.” On one hand, activated microglia/macrophages aggravate neuroinflammation through antigen presentation and proinflammatory cytokine secretion [123]. Microglia/macrophages can directly damage neurons by releasing inflammatory factors (reactive oxygen and nitrogen species) to trigger mitochondrial injury and axonal damage [124]. On the other hand, microglia/macrophages drive oligodendrocyte differentiation and initiate remyelination. Microglia can also clear myelin debris and apoptotic cells as well as promote neurogenesis by producing neurotrophic factors such as brain-derived neurotrophic factor (BDNF) and insulin-like growth factor 1 (IGF-1) [125].

**Fig. 6:**
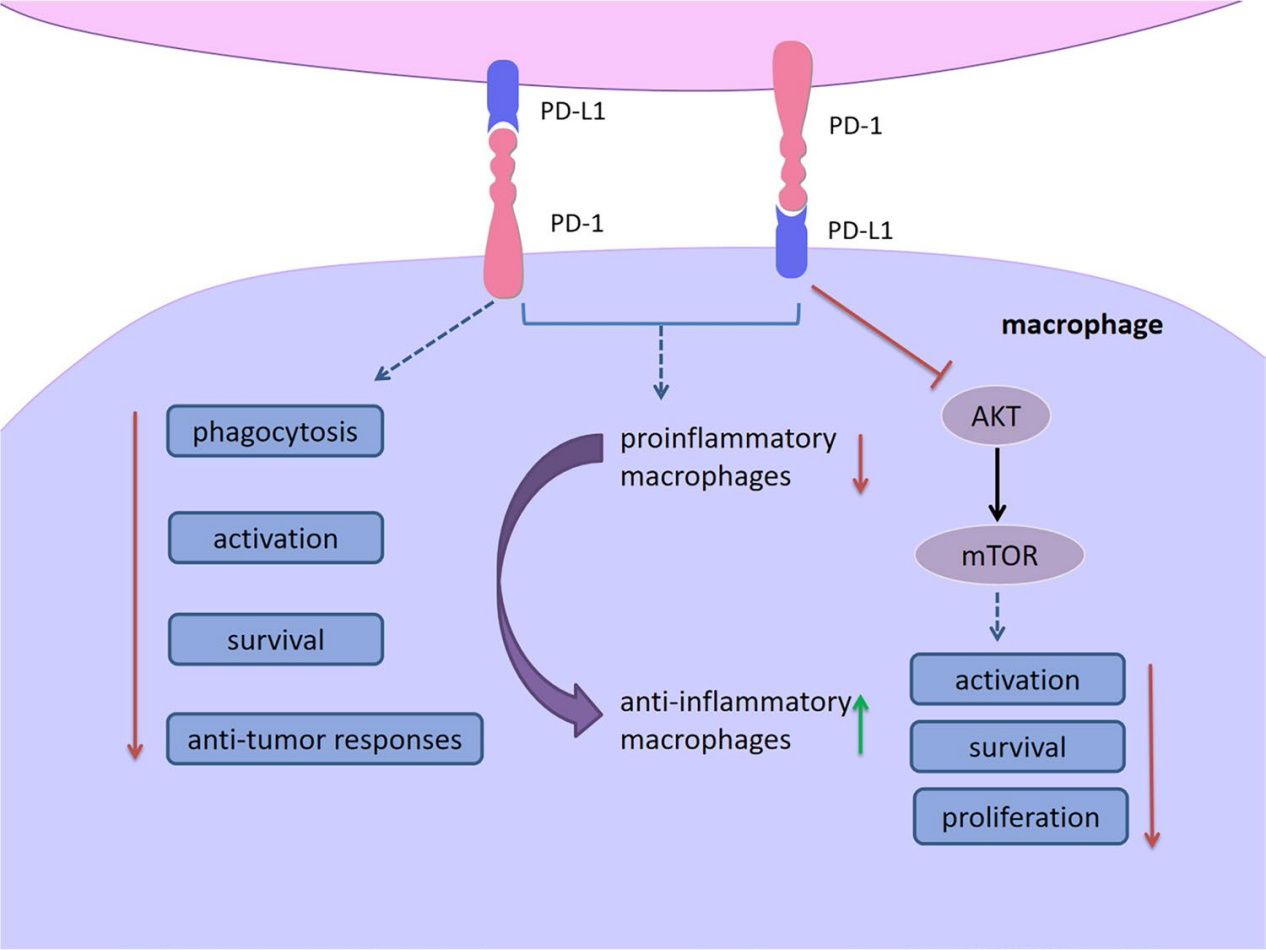
PD-1/PD-L1 signaling in macrophages. PD-1-expressing macrophages exhibit an anti-inflammatory-like surface profile in tumor settings. PD-1 expression is negatively correlated to phagocytic ability of macrophages and blockade of PD-1/PD-L1 enhanced anti-tumor responses, prolonged survival through inhibiting proinflammatory to anti-inflammatory macrophages polarization. PD-L1 antibody treatment promotes cell proliferation of cultured bone marrow-derived macrophages, which is related to the activation of the AKT-mTOR pathway. Transcriptomic profiles of macrophages have been switched into inflammatory phenotypes following PD-L1 antibody treatment. *Abbreviations*: PD-1, programmed cell death 1; PD-L1, programmed cell death 1 ligand 1

PD-1-expressing macrophages exhibited an anti-inflammatory-like surface profile in both mice and human tumor settings [126]. PD-1 expression is negatively correlated to phagocytic ability of macrophages and blockade of PD-1/PD-L1 enhanced anti-tumor responses, prolonged survival through inhibiting proinflammatory to anti-inflammatory macrophages polarization [126, 127]. PD-L1 antibody treatment promoted cell proliferation of cultured bone marrow-derived macrophages, which is related to the activation of the AKT-mTOR pathway. Transcriptomic profiles of macrophages have been switched into inflammatory phenotypes following PD-L1 antibody treatment [128] (Fig. 6).

**Fig. 7:**
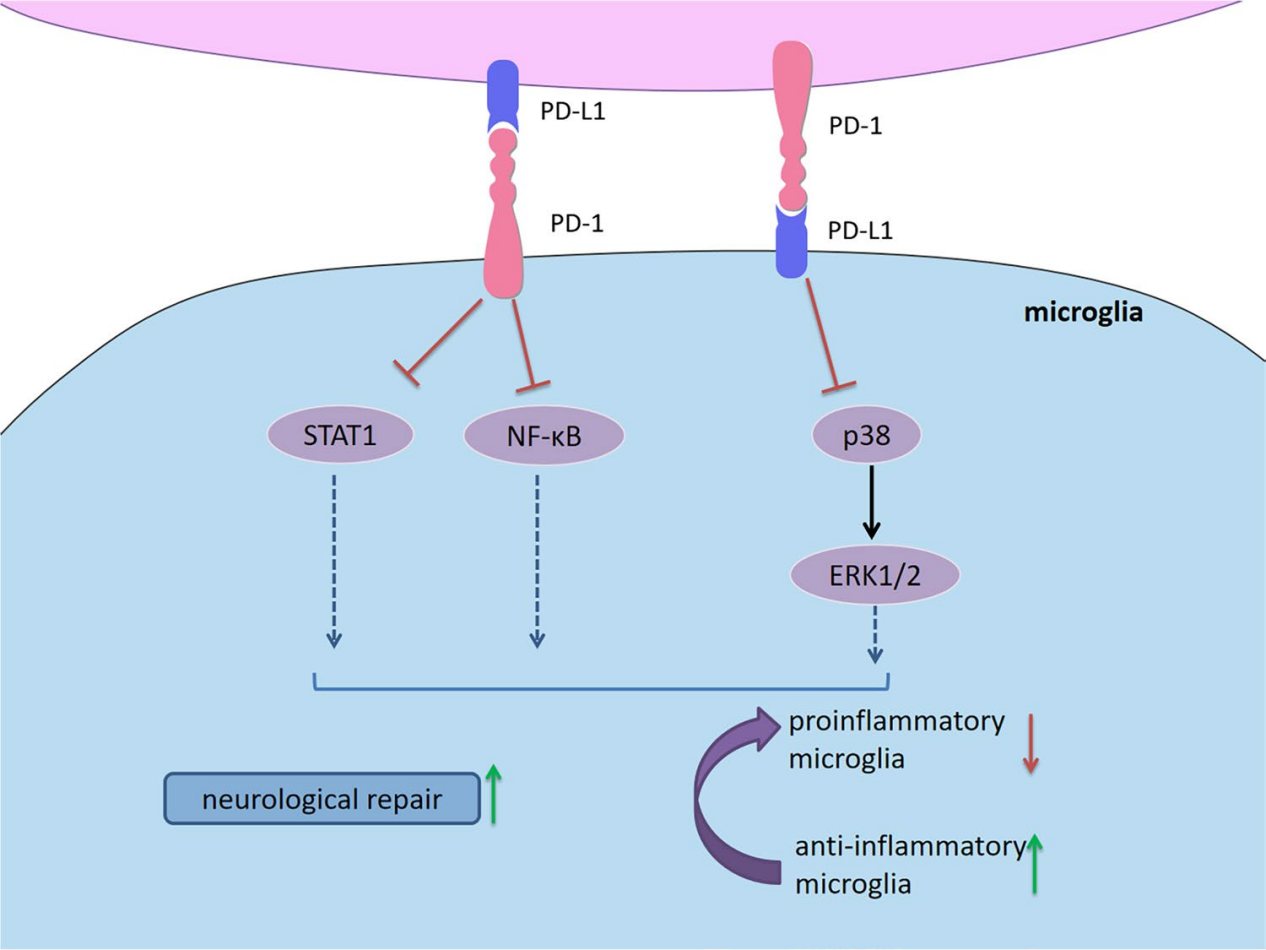
PD-1/PD-L1 signaling in microglia. During neuroinflammation, microglia upregulate the expression of both PD-1 and PD-L1. Upregulated expression of PD-1 in activated microglia can reduce proinflammatory cytokine production, inducing microglial polarization toward into the immunoregulatory type. In mice model of spinal cord injury, PD-1 deficiency induces microglial polarization toward into the proinflammatory phenotype via STAT1 and NF-κB signaling. The elevated expression of PD-L1 promotes anti-inflammatory microglial polarization via inhibiting the phosphorylation of p38 and ERK1/2. With the upregulation of PD-1 on infiltrating effector T cells in the CNS, microglia are highly expressed PD-L1 and suppress T-cell responses via the PD-1/PD-L1 interactions, limiting detrimental immune-mediated damage. Abbreviations: PD-1, programmed cell death 1; PD-L1, programmed cell death 1 ligand 1; NF-κB, nuclear factor kappa beta; ERK, extracellular signal–regulated kinase

During neuroinflammation, microglia upregulate the expression of both PD-1 and PD-L1, serving as a critical immune cell type that attenuates inflammatory responses and promotes neuronal repair. Upregulated expression of PD-1 in activated microglia can reduce proinflammatory cytokine production, inducing microglial polarization toward into the immunoregulatory type [129]. In mice model of spinal cord injury, PD-1 deficiency induced microglial polarization toward into the proinflammatory phenotype via STAT1 and nuclear factor kappa beta (NF-κB) signaling [130]. It was recently discovered that the elevated expression of PD-L1 promoted anti-inflammatory microglial polarization after spinal cord injury, then improving motor function recovery and alleviating neuropathic pain via inhibiting the phosphorylation of p38 and ERK1/2 [131]. With the upregulation of PD-1 on infiltrating effector T cells in the CNS, microglia are highly expressed PD-L1 and suppress T-cell responses via the PD-1/PD-L1 interactions, limiting detrimental immune-mediated damage [132]. Since the expression of PD-1 and PD-L1 can be upregulated on microglia simultaneously, the possibility of microglia interacting with themselves through the PD-1/PD-L1 pathway is worth exploring in the future (Fig. 7).

Taken together, the expression of PD-1 and PD-L1 on microglia/macrophages occurs as a response in the presence of different inflammatory milieu, altering cellular phenotypes through downstream signal transduction, then modulating innate and adaptive immune responses.

## Current Understanding of PD-1/PD-L1 in MS/EAE

**Fig. 8:**
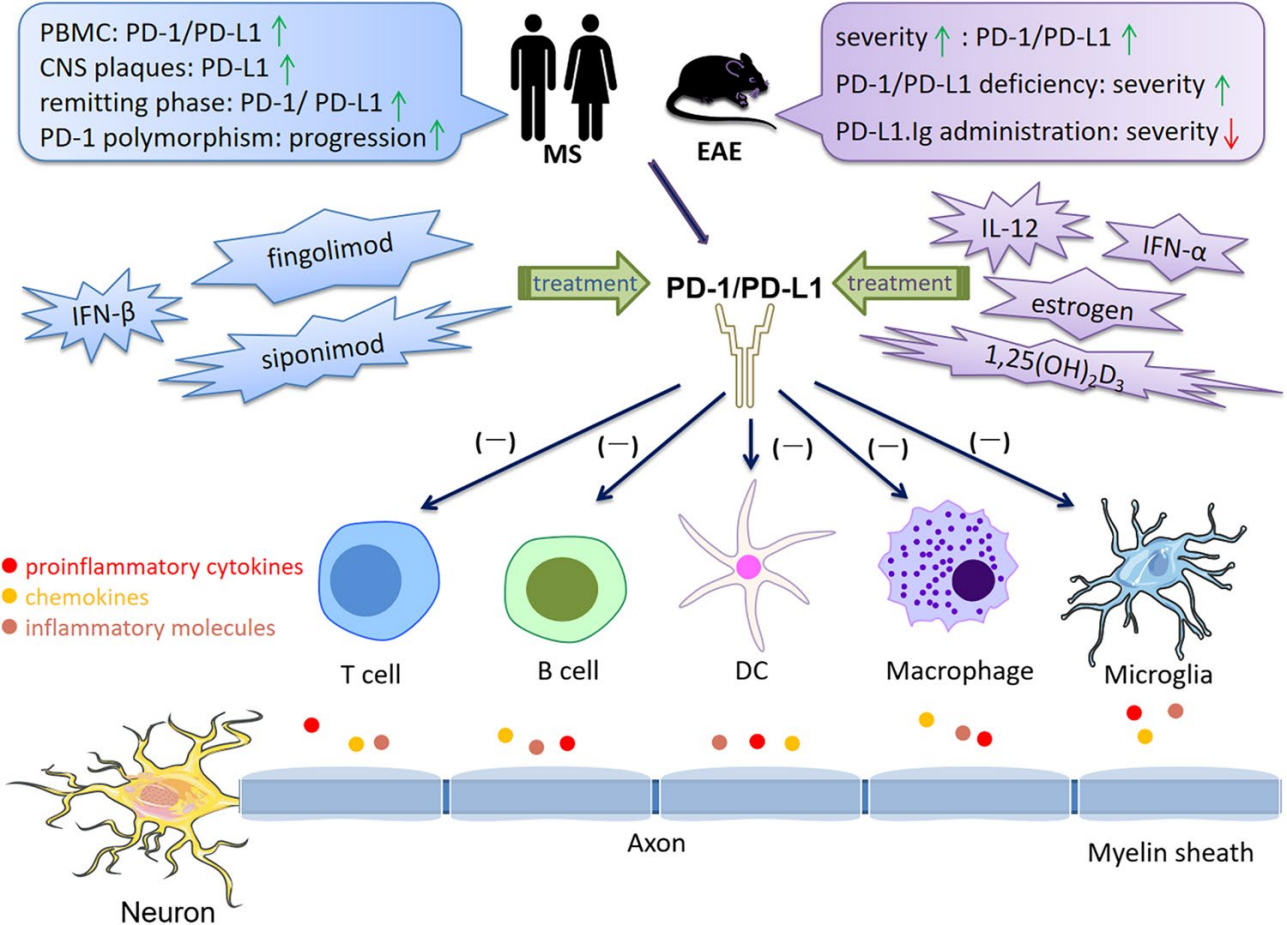
Cell-based PD-1/PD-L1 immunoregulation in MS/EAE. There is a correlation between PD-1/PD-L1 expression and initiation along with disease progression. Compared to healthy individuals, the expression of PD-1/PD-L1 on PBMCs was significant reduced in RRMS patients. While PD-L1 expression on MS plaques was increased, PD-1-expressing T cells and PD-L1-expressing APCs in peripheral blood of remitting MS patients were significantly increased compared with acute MS patients. Importantly, an intronic 7146G/A polymorphism within the *Pdcd1* gene is associated with a progressive disease course in MS. In EAE, the expression of PD-1/PD-L1 was increased with worsening symptoms. Blockade or deficiency of PD-1/PD-L1 resulted in EAE exacerbation. Early treatment with PD-L1Ig fusion protein resulted in a long-lasting disease amelioration. Furthermore, upregulation of PD-1/PD-L1 is involved in the immunoregulation of many treatments for MS/EAE, including IFN-β, siponimod, fingolimod, IFN-α, IL-12, estrogen and 1,25(OH)_2_D_3_. T cells (Th1, Th17, CTLs), B cells, DCs, and macrophages/microglia play pathogenic role in MS/EAE. They produce proinflammatory cytokines, chemokines and molecules in the CNS, causing myelin injury, axonal damage and neuron loss. Upregulation of PD-1/PD-L1 inhibits over-reactive immune responses through inducing immunoinhibitory or tolerogenic immune cells, restricting neuroinflammation in MS/EAE. Furthermore, PD-1 and PD-L1 have been identified to be involved in the therapeutic responses for MS/EAE. *Abbreviations*: PD-1, programmed cell death 1; PD-L1, programmed cell death 1 ligand 1; MS, multiple sclerosis; EAE, experimental autoimmune encephalomyelitis; PBMC, peripheral blood mononuclear cell; RRMS, relapsing–remitting multiple sclerosis; APC, antigen-presenting cell; IFN, interferon; IL, interleukin; 1,25(OH)_2_D_3_, 1,25-dihydroxyvitamin D3; CTL, cytotoxic T lymphocytes; DC, dendritic cell; CNS, central nervous system

Given that PD-1/PD-L1 emerges as a significant player in immune tolerance, the role of PD-1/PD-L1 signaling in MS/EAE has received increasing attention. This section summarizes the role of PD-1/PD-L1 in the conditions of MS/EAE (Fig. 8).

Compared to healthy individuals, the expression of *pd-1* and *pd-l1* in peripheral blood mononuclear cells (PBMCs) was significantly reduced in RRMS patients, suggesting the breakdown of immunological tolerance in MS [133, 134]. However, PD-L1 expression in MS plaques has been shown to be increased when compared to non-pathological human CNS tissues [135]]. Upon myelin basic protein (MBP) stimulation, PD-1-expressing T cells and PD-L1-expressing APCs in peripheral blood of remitting MS patients were significantly increased compared with acute MS patients, indicating a positive correlation between PD-1/PD-L1 expression and MS remission [136]. A potential explanation for these paradoxical results may be the heterogeneity of patients’ baseline characteristics and disease course in these studies. Importantly, PD-1 polymorphism is suggested to be a genetic modifier of MS progression [47]. An intronic 7146G/A polymorphism of *Pdcd1* gene is associated with a progressive disease course in German MS patients by impairing PD-1-mediated suppression of IFN-γ secretion [47]. A latest prospective, longitudinal study measured the serum PD-L1 during pregnancy and postpartum in MS and the results showed that there was a trend of increased PD-L1 during the first trimester of pregnancy in patients without disease relapse, but did not achieve statistical significance [137]. In EAE, PD-1/PD-L1 can be expressed on infiltrating mononuclear cells within the meninges and PD-L1 is expressed on the endothelium, astrocytes and microglia [138, 139]. The expression of PD-1 and PD-L1 was significantly increased with disease progression [139]. Blockade of PD-1 using neutralizing mAb resulted in disease exacerbation, associated with increased myelin oligodendrocyte glycoprotein (MOG)-reactive T-cell responses, anti-MOG antibody production and CNS lymphocyte infiltration [139]. In keeping with this, EAE mice with the deficiency of PD-1 and PD-L1 developed severe disease [22]. In active MOG-induced EAE, early treatment with PD-L1Ig fusion protein resulted in a long-lasting disease amelioration [140]. Collectively, these discoveries highlight a protective role of PD-1/PD-L1 in EAE.

The number of memory Tregs are reduced expressing significantly high level of PD-1 in peripheral blood of RRMS patients [141]. Liu and colleagues identified a novel Treg subpopulation (CD4^+^FoxA1^+^CD47^+^CD69^+^PD-L1^hi^FoxP3^−^) in EAE and adoptive transfer of these Tregs suppressed EAE depending on the PD-L1 expression [142]. Furthermore, PD-L1 deficiency enhanced Th1 and Th17 responses in EAE, and the numbers of CD4^+^ and CD8^+^ T cells in the CNS were significantly elevated [135]. Proinflammatory microRNA (miR)-155 is critical for T cell effector functions with miR-155-deficient mice being highly resistant to EAE due to the Th1 and Th17 differentiation defect. A recent study found that PD-1 deletion promotes EAE in miR-155-knockout mice by increasing Th1 and Th17 cell infiltration [42]. Adoptive transfer of Bregs, which highly express PD-L1, can suppress the incidence and severity of MOG-induced EAE by decreasing IFN-γ and IL-17 production [98]. Estrogen is believed to engage in immunomodulation and contributes to disease protection in MS/EAE. PD-L1 selectively expressed by B cells was proven to be indispensable for estrogen-induced protection against EAE [143]. Nonetheless, depletion of PD-L1 on B cells did not affect the onset and severity of EAE [144]. When EAE was induced in DC-depleted mice, a high degree of inflammation was observed, indicating the capacity of DCs for peripheral tolerance [119]. Mechanistic experiments indicated that the interactions between DCs and T cells by PD-L1/PD-1 ligation led to T cell hyporesponsiveness to EAE [119]. Consistently, conditional knockout of PD-L1 in DCs aggravated EAE [144]. The upregulation of PD-L1 in DCs via DNA hypomethylation resulted in delayed progression of EAE [145], and tolerogenic DCs expressed a high level of PD-L1 were effective for EAE [118, 146]. However, Zozulya et al. found that intracerebral injection of PD-L1^−/−^ DCs recruited regulatory CD8^+^ T cells into the CNS and then ameliorated EAE [147]. It seems that PD-L1 on DCs is pivotal to maintain the susceptibility and reactivity of both pathogenic and protective T cells in EAE.

PD-L1 on microglia/macrophages plays a role in restricting neuroinflammation. Estrogen treatment enhanced PD-L1 expression on peripheral macrophages, which may contribute to its efficacy for EAE [148]. Previous studies revealed that PD-L1 on microglia can inhibit antigen-specific T cell activation, Th1 differentiation and cytokine production in vitro [132, 149]. PD-L1 expression on microglia/infiltrating macrophages has been shown to be increased in EAE. The role of PD-1/PD-L1 on NK cells in MS/EAE remains unclear. It is well documented that PD-1/PD-L1 signaling regulates the functions of NK cells playing a crucial role on MS pathology. More efforts are needed to explore PD-1/PD-L1 on NK cells as a potential target in the future.

PD-1 and PD-L1 have been identified to be involved in therapeutic mechanisms of MS/EAE. Both short- and long-term injection of IFN-β upregulated the expression of PD-L1 in PBMCs of RRMS patients [150, 151]. A randomized controlled trial of siponimod (a selective sphingosine-1-phosphate receptor 1 and 5 modulator) indicated that treating SPMS with siponimod for 9–12 months increased circulating Tregs proliferation and upregulated PD-1 expression on CD4^+^ T cells [152]. Similarly, PD-1 expression on Tfh cells in the peripheral blood was increased following fingolimod treatment (12-month) in MS (153). Sustained low-dose IFN-α showed prophylactic and therapeutic efficacy in EAE by upregulating the mRNA expression of PD-1 on splenocytes (154). IL-12 administration can suppress EAE by increasing PD-L1 expression on CD11b^+^ APCs via an IFN-γ-dependent manner [155]. Several studies have unraveled that mechanisms of estrogen to prevent EAE also include the upregulation of PD-1/PD-L1 [143, 146, 148, 156]. We have previously demonstrated that tolerogenic DCs induced by 1,25-dihydroxyvitamin D3 (1,25(OH)_2_D_3_) are effective for EAE and potential therapeutic effects can be related to increased expression of PD-1 on T cells [118]. In summary, PD-1 and PD-L1 may serve as a promising target for MS/EAE.

## Mechanisms of Regulating PD-1/PD-L1 Expression

Since PD-1/PD-L1 ligation plays a role in maintaining immune homeostasis and tolerance, a better understanding of biological processes that regulate PD-1 and PD-L1 expression is helpful. The expression of PD-L1 and PD-1 can be tightly regulated via a complex manner, involving different signaling pathways, genomic and epigenetic parameters (summarized in Table 2).

**Table 2:** Regulation of PD-L1 and PD-1 expression

	Cytokines		Signaling pathways		Transcriptional factors		Epigenetic parameters	
	Inducer (↑)	Repressor (↓)	Inducer (↑)	Repressor (↓)	Inducer (↑)	Repressor (↓)	Inducer (↑)	Repressor (↓)
PD-L1	IFN-γ (160), TNF-α (161), IL-17 (162), IFN-α (163), IFN-β (163), IL-2 (164), IL-7 (164), IL-15 (164), IL-21 (164), IL-27 (165), IL-18 (166), IL-6 (29), IL-1β (167), IL-10 (168)		JAK1/JAK2-STAT1/STAT2/STAT3 (160), PI3K-AKT (169), MEK-ERK (169), TLRs-NF-κB (171)	PTEN (182)	IRF-1 (160), HIF-1α (172)		MiR-20b (182), miR-21 (182), miR-130b (182)	MiR-513 (176), miR-155 (177), miR-152 (178), miR-200b (178), miR-15b (179), miR-16 (179), miR-193a-3p (179), miR-195 (179), miR-200c (179), miR-142-5p (180), miR-197 (181)
PD-1	IL-2 (164), IL-7 (164), IL-15 (164), IL-21 (164), IFN-α (183–185), IL-6 (185), IL-12 (185,186), IL-18 (186),	IL-4 (121)	TLR2 (121), TLR3 (121), TLR4 (121), NF-κB (187), calcineurin (188), STAT3 (185), STAT4 (185), JAK1-STAT1/STAT2 (183, 184), PKC-RAS (189)	PI3K-AKT (185)	NFATc1 (188,191), ISGF3 (183,184), AP-1 (189), FoxO1 (190), Bcl-6 (193)	Blimp-1 (191), T-bet (192)	Histone acetylation (188), histone trimethylation (188), DNA demethylation (194)	DNA methylation (194), miR-28 (195), miR-138 (196)

*Abbreviations*: *IFN*, interferon; *TNF*, tumor necrosis factor; *IL*, interleukin; *JAK*, janus kinase; *STAT*, signal transducer and activator of transcription; *PI3K*, phosphatidylinositol 3-kinase; *MEK*, extracellular signal-regulated kinase kinase; *ERK*, extracellular signal-regulated kinase; *TLRs*, Toll-like receptors; *NF-κB*, nuclear factor kappa beta; *PTEN*, phosphatase and tensin homolog; *IRF-1*, interferon regulatory factor-1; *HIF-1α*, hypoxia-inducible factor-1α; *miR*, microRNA; *PKC*, protein kinase C; *RAS*, retrovirus-associated DNA sequences; *NFATc1*, nuclear factor of activated T cells c1; *ISGF3*, IFN-stimulated gene factor 3; *AP-1*, activator protein 1; *Bcl*, B-cell lymphoma; *Blimp-1*, B lymphocyte–induced maturation protein 1; *T-bet*, T-box expressed in T cells

### Regulation of PD-L1 Expression

Expression of *pd-l1* gene has been shown to be controlled by inflammatory milieu. Multiple proinflammatory cytokines are prominent soluble inducers for PD-L1, including TNF-α, IL-17, IFN-α, IFN-β, IFN-γ, IL-2, IL-7, IL-15, IL-21 and IL-27 [160–165]. IL-18 derived from cancer cells can promote PD-L1 expression and IL-10 production [166]. IL-6, IL-1β, TNF-α, IL-10, and IL-27 increase PD-L1 expression on DCs [29, 167, 168]. IFN-γ-mediated PD-L1 expression is mainly regulated by janus kinase 1 (JAK1)/JAK2-STAT1/STAT2/STAT3, PI3K-AKT, MEK-ERK and NF-κB pathways and transcription factor interferon regulatory factor-1 (IRF-1) [160, 169, 170]. Notably, the activation of PI3K-AKT and MEK-ERK can augment PD-L1 expression, while PD-1 signaling negatively regulates these two pathways decreasing PD-L1 expression and inhibiting T cell functions [10, 71, 167]. It is a negative feedback loop of immune cells to respond to microenvironment changes. Signal transduction via pathogen-associated molecular patterns (PAMPs) and Toll-like receptors (TLRs) results in nuclear translocation of NF-κB, binding NF-κB to the PD-L1 promoter and inducing PD-L1 expression [171]. In the condition of hypoxia, hypoxia-inducible factor-1α (HIF-1α) binds to the hypoxia-response element (HRE) in the PD-L1 proximal promoter and cooperates with the NF-κB pathway to promote PD-L1 transcription [172]. Besides, NF-κB also enhances PD-L1 protein stability [173]. There are paradoxical findings about the PD-L1 regulation by NF-κB. Inhibiting NF-κB pathway can induce tolerogenic DCs, which express higher level of PD-L1 than untreated-DCs [174, 175]. The diversity of PD-L1 regulation by NF-κB pathway in different immune cell types and the crosstalk among different signaling pathways may contribute to the discordance. MiRNAs function as post-transcriptional and translational regulators of PD-L1 expression [176–181]. Specifically, miR-20b, miR-21 and miR-130b can enhance PD-L1 expression by inhibiting the expression of PTEN, which is correlated with the activation of PI3K-AKT [182].

### Regulation of PD-1 Expression

IL-2, IL-7, IL-15, IL-21, IFN-α, IL-6, IL-12 and IL-18 were reported to promote the expression of PD-1 in different immune cells [164, 183]. IL-4 exerts negative effects on PD-1 expression in mice DCs, while IL-2, IL-6, IL-10, IFN-γ, IL-12, and TNF-α displayed no significant influences [121]. The upregulation of PD-1 on B cells can be induced by TLR9 activation [95]. However, TLR9 significantly inhibits PD-1 expression on mice DCs, whereas TLR2, TLR3 and TLR4 induce PD-1 expression on DCs [121]. Following lipopolysaccharide (LPS) stimulation, PD-1 expression of macrophages can be induced by the activation of NF-κB [187]. Upon TCR stimulation of CD8^+^ T cells, calcineurin pathway is activated, resulting in translocation of nuclear factor of activated T cells c1 (NFATc1). NFATc1 binded to the conserved region-c (CR-C) enhances the promoter activity of PD-1 [188]. IL-6 and IL-12 can enhance PD-1 transcription on activated T cells by binding STAT3 and STAT4, respectively [185]. As for IFN-α-induced PD-1 expression, the binding of IFN-α to its receptor leads to the activation of JAK1-STAT1/STAT2 pathway. STAT1, STAT2, and cytosolic IFN-responsive factor 9 (IRF9) proteins then form a transcription complex called IFN-stimulated gene factor 3 (ISGF3), which binds to the interferon-sensitive responsive element (ISRE) motif in the PD-1 promoter [183,184]. Upon TCR stimulation, activator protein 1 (AP-1) is upregulated due to the PKC-RAS activation. AP-1 subunit c-Fos directly binds to the AP-1 site in the CR-B region of the *Pdcd1* locus and increase PD-1 expression [189]. As discussed before, PD-1 signaling dampens TCR-dependent activation of PI3K-AKT pathway. A previous study demonstrated that the inhibition of PI3K-AKT can enhance the nuclear accumulation of the transcription factor FoxO1, which reinforces PD-1 expression [190]. The results highlight a positive feedback pathway of PD-1 expression, promoting T cell exhaustion and immune homeostasis during chronic infection. The binding of B lymphocyte–induced maturation protein 1 (Blimp-1) in *Pdcd1* gene lucos leads to the eviction of NFATc1 from its site and the formation of a repressive chromatin structure, inhibiting the expression of PD-1 [191]. Additionally, Blimp-1 can curtail PD-1 expression indirectly by repressing the expression of NFATc1 [191]. During chronic infection the expression of T-box expressed in T cells (T-bet) can be downregulated in exhausted CD8^+^ T cells (highly express PD-1), while the over-expression of T-bet reduces the expression of PD-1 through binding to upstream regulatory elements of *Pdcd1* [192]. In accordance with this, transcription factor Bcl-6, acting as a PD-1 stimulator in Tfh cells, can inhibit the ability of T-bet to repress PD-1 transcription [193]. Epigenetic factors including histone modifications, DNA methylation and miRNAs can also affect the expression of PD-1 [188,194]. However, DNA demethylation in CR-C and CR-B regions is not indispensable for PD-1 expression in immune cells, while may be important for durable PD-1 expression. It has been indicated that miR-28 and miR-138 can reduce the PD-1 expression on T cells [195,196], while the knowledge about regulating PD-1 expression by miRNAs remains insufficient. More research in this area is needed to fill the gaps.

## Concluding Remarks and Future Directions

PD-1 and PD-L1 act as the brake of immune system, protecting self-tissue from autoimmunity. PD-1/PD-L1 strikes a balance between protective immunity and immunopathology by regulating the functions of immune cells. Exploring the precise effects and mechanisms of PD-1/PD-L1 on each cell type in certain immune microenvironments is of vital importance for clinical application of PD-1/PD-L1 targeting therapies. The functions of PD-1/PD-L1 are not completely overlapping [128, 140] and the underlying diversity needs to be further explored. Apart from its expression on immune cells, tissue expression of PD-1/PD-L1 also plays an immunoregulatory role [138, 197], which should be concerned in autoimmune diseases. The interactions between PD-1/PD-L1 signaling and cellular metabolism have been indicated recently [198], providing a new promising research direction of PD-1/PD-L1.

A wave of studies regarding PD-1/PD-L1 in cancer and autoimmune diseases has provided valuable evidence for understanding the role of PD-1/PD-L1 in MS/EAE. Dynamic PD-1/PD-L1 expression during MS is inconclusive. To resolve the dispute, detailed baseline characteristics, clinical classifications and disease courses of MS should be taken into consideration. Most researches focused on the regulation of PD-1 on T cell activation in MS/EAE. Due to the involvement of B cells, NK cells, DCs, microglia/macrophages in MS pathology, the expression of PD-1/PD-L1 on these cells may also engage in MS development. Specifically, PD-1/PD-L1 not only limits autoimmunity in the CNS, but also exerts influences on acute and chronic pain, affective and cognitive behaviors [199, 200]. Therefore, additional physiological functions of PD-1/PD-L1 in the CNS may provide new insights in MS pathogenesis. Based on current literature of PD-1/PD-L1 in MS/EAE, we conclude that PD-1/PD-L1 may be useful in indicating disease progression, monitoring therapeutic responses and predicting prognosis in MS, while the reliability as molecular biomarkers should be further validated.

Targeting PD-1/PD-L1 by direct administration of agonists or indirect upregulated expression may be potential specific treatments for MS, mirroring success of PD-1/PD-L1 reinforcement in the treatment of autoimmunity. In order to design therapeutic strategies safely and effectively, a variety of open questions need to be addressed: [1] Which cell types should be targeted by PD-1/PD-L1 enhancement? Given that PD-1 and PD-L1 are both expressed on various immune cells, we cannot exclude the possibility of bidirectional interactions between PD-1 and PD-L1 on these cell types. Conditional knockout of PD-1/PD-L1 in certain immune cell types may be helpful. [2] What are the appropriate time, treatment regimens, and patients for PD-1/PD-L1 reinforcement? Due to the heterogeneity of disease course and therapeutic responses in different patients with MS, the best intervention timepoint, the feasible combination therapeutics regimens and selective patients who may benefit from PD-1/PD-L1-based therapies should be confirmed to achieve an optimized therapeutic outcome. [3] PD-1/PD-L1 therapies used for alleviate neuroinflammation must have superior bioavailability across the BBB. The ideal carrier can overcome the obstacles and deliver PD-1/PD-L1-based drugs into the CNS. [4] Are there potentially adverse effects of PD-1/PD-L1 therapies? The overwhelming anti-tumor efficacy of PD-1/PD-L1 blockade is usually accompanied by immune-related adverse events [201]. PD-1/PD-L1 signaling plays a role in maintaining self-tolerance and preventing autoimmunity through general immune suppression, detrimental off-target effects may impair viral clearance, cause long-drawn-out opportunistic infections, and even increase the risk of cancer. Therefore, the negative impact of PD-1/PD-L1 reinforcement on the motility of immune surveillance and immune defense during long-term period should be thoroughly assessed. Identifying potential patients who will suffer from adverse effects following PD-1/PD-L1 administration may guide researchers to find a way to minimize these possible adverse events.

In conclusion, PD-1/PD-L1 plays an immunoregulatory role in a variety of immune cells including T cells, B cells, NK cells, DCs and macrophages/microglia in MS/EAE. PD-1/PD-L1 negatively regulates immune response. Furthermore, PD-1/PD-L1 is actively involved in therapeutic efficacy of current DMTs for MS. Updated knowledge of PD-1/PD-L1 provides theoretical foundation for manipulating PD-1/PD-L1 signaling pathway as a promising therapeutic target for MS in the future.

## Data Availability

Not applicable.
